# Alternative Lengthening of Telomeres is characterized by reduced compaction of telomeric chromatin

**DOI:** 10.1093/nar/gku114

**Published:** 2014-02-05

**Authors:** Harikleia Episkopou, Irena Draskovic, Amandine Van Beneden, Gaëlle Tilman, Marina Mattiussi, Matthieu Gobin, Nausica Arnoult, Arturo Londoño-Vallejo, Anabelle Decottignies

**Affiliations:** ^1^Genetic and Epigenetic Alterations of Genomes, de Duve Institute, Catholic University of Louvain, Brussels 1200, Belgium and ^2^Telomeres and Cancer Laboratory, Equipe Labellisée Ligue, UMR3244-UPMC-Institut Curie, Paris 75248, France

## Abstract

Proper telomeric chromatin configuration is thought to be essential for telomere homeostasis and stability. Previous studies in mouse suggested that loss of heterochromatin marks at telomeres might favor onset of Alternative Lengthening of Telomeres (ALT) pathway, by promoting homologous recombination. However, analysis of chromatin status at human ALT telomeres has never been reported. Here, using isogenic human cell lines and cellular hybrids, which rely either on telomerase or ALT to maintain telomeres, we show that chromatin compaction is reduced at ALT telomeres and this is associated with a global decrease in telomeric H3K9me3. This, subsequently, leads to upregulation of telomere transcription. Accordingly, restoration of a more condensed telomeric chromatin through telomerase-dependent elongation of short ALT telomeres reduces telomere transcription. We further show that loss of ATRX chromatin remodeler function, a frequent characteristic of ALT cells, is not sufficient to decrease chromatin condensation at telomeres nor to increase the expression of telomeric RNA species. These results offer new insight on telomeric chromatin properties in ALT cells and support the hypothesis that telomeric chromatin decondensation is important for ALT pathway.

## INTRODUCTION

Telomeres are specialized structures that protect the ends of chromosomes from degradation and fusion (1). Unlimited replication potential is conferred to cells that activate a telomere maintenance mechanism (TMM). This TMM is dependent on either telomerase, a reverse transcriptase adding telomeric repeats at chromosome ends, or on one or more so-called ‘ALT’ (Alternative Lengthening of Telomeres) mechanism(s), still poorly understood but known to rely on telomeric homologous recombinations (2). Approximately 10–15% of all human tumors do not express telomerase (3). Although not frequently detected in epithelial malignancies, the ALT phenotype is prevalent in some sarcoma subtypes, in astrocytomas and glioblastomas. ALT cells are characterized by heterogeneous telomere lengths, ranging from undetectable to >50 kb, and by the presence of extrachromosomal telomeric DNA molecules that accumulate within ALT-associated Promyelocytic leukemia (PML) bodies (APBs) (2). Owing to recombination events with upstream subtelomeric sequences, ALT telomeres are also characterized by the presence of variant repeat sequences, mostly of the C-type (TCAGGG) (4). These repeat variants offer binding sites for orphan receptors, whose function at ALT telomeres remains to be determined (4,5). With respect to emerging anti-cancer therapies targeting telomere maintenance, it is crucial to get a better understanding of ALT mechanism (6). In this view, identifying structural differences between telomerase- and ALT-dependent telomeres is likely to provide useful information.

Telomeres are organized in regularly spaced and tightly packed nucleosomes, with linker DNA being ∼40 bp shorter compared with the bulk chromatin (7,8). Studies in mouse and human cells also revealed that telomeric chromatin is enriched in marks associated with constitutive heterochromatin (HC), such as H3K9me3 and H4K20me, HP1 accumulation and histone hypoacetylation (9–12). Consistently, human SIRT6 was reported to deacetylate telomeric H3K9 (11). We also recently showed that the enrichment of both H3K9me3 and HP1 at human telomeres is cell cycle–regulated and is increased at longer telomeres, suggesting the existence of tightly regulated mechanisms for telomeric HC formation (12).

Various findings led to the proposal that reduction of telomeric HC marks may favor ALT mechanism by promoting telomeric recombinations. First, depletion of mouse Suv39h or Suv4-20 h histone methyltransferases leads to telomere elongation, associated with increased telomeric recombination and the appearance of APBs (9,10,13). Second, downregulation of either HDAC5 histone deacetylase (14) or NoRC/TIP5 (15), a chromatin remodeling complex involved in HC formation, increases telomeric recombination frequency in ALT cells. Finally, although the underlying mechanisms are not yet understood, ALT phenotype in various types of tumors and cell lines has been correlated with the loss of X-linked ATRX chromatin remodeler expression (16–18).

In addition to a possible impact on recombination events, telomeric HC marks regulate transcriptional activity at telomeres and thus influence the cellular amount of telomeric repeat-containing RNAs (TERRA) (12). TERRA molecules remain partly associated with telomeres (12,19–21) where they are likely to fulfill important functions, including HC formation (22), control of telomerase activity (23), cell cycle–regulated telomeric loop folding (24) and telomerase recruitment (25). On the other hand, telomeric RNA/DNA heteroduplexes may favor replication fork collapses and activate homologous recombination at telomeres (26). Interestingly, telomere transcription *per se* may also promote telomeric recombination by hampering replication fork progression (27).

The above data prompted us to assess chromatin status and transcriptional activity at ALT telomeres through comparative analysis of ALT and telomerase-expressing (TEL+) fibroblasts with similar genetic backgrounds. Our results indicated that, when compared with TEL+ cells, chromatin of ALT telomeres is more relaxed and associated with increased TERRA expression.

## MATERIALS AND METHODS

### Cell lines

SW39 (GenetR) and IMRB cell lines were described previously (28). For selection purposes, IMRB cells were transfected with pBABE::Hygro plasmid. Cellular hybrids between SW39 and IMRBHygroR were obtained by incubation of 2 × 10^6^ cells of each parent with 0.9 g/ml PEG/10% dimethyl sulfoxide (DMSO) for 1 min. Hybrids were selected with 0.2 mg/ml hygromycin (Sigma) and 2 mg/ml geneticin (Thermo Fisher) for 2 weeks before sub-cloning. IMRB-Telo15 cell line was obtained by transfecting IMRB cells first with pBabe::U1-hTR-hygro plasmid (28) and then, after selection with 0.2 mg/ml hygromycin, with pBMN::hTERT-puro (kindly provided by C Heirman, VUB, Brussels). IMRB-Telo7 was another clone isolated after transfection with pBMN::hTERT-puro and selection with 0.6 μg/ml puromycin. To get shATRX-1, -2 and -3 clones, SW39 cells were transfected with an ATRX-specific shRNA construct (Sigma, clone 592) targeting CCGGTGGTGAACATAAGAAAT. Cells were selected with puromycin (0.6 μg/ml) for 4 days, sub-cloned and at least 2 × 10^6^ cells of each clone were maintained in culture for 110 days. Transfection of SW39 cells with the empty plasmid was used as a negative control to generate three control clones.

### Fluorescence *in situ* hybridization

Quantitative fluorescence *in situ* hybridization (FISH) was carried out as described previously, using Cy3-O-O-(CCCTAA)_3_ PNA probe (Panagene) (29). A detailed protocol is provided in Supplementary methods. Images were acquired using Zeiss Axioplan 2. Telomeric signals were quantified with iVision software (Chromaphor) and corrected for the average local background. Statistical analyses were done using the Wilcoxon rank-sum test.

### Quantitative telomerase repeat amplification protocol

Two hundred fifty nanograms of whole-cell extracts in CHAPS were assayed by quantitative telomerase repeat amplification protocol (qTRAP) using GoTaq qPCR Master Mix (Promega) and both TS (5′-AATCCGTCGAGCAGAGTT-3′) and ACX (5′-GCGCGGCTTACCCTTACCCTT-ACCCTAACC-3′) primers at 200 nM. Cycles were as follows: 25°C for 30 min, 95°C for 5 min and 40 cycles with 95°C for 30 s, 60°C for 30 s and 72°C for 1 min. Specificity of TRAP reaction was checked on EtBr-staind 20% precast TBE gel (Invitrogen).

### Western blot and immunofluorescence

Western blots and immunofluorescence were performed as described previously (12) using antibodies listed in Supplementary Table S1.

### Telomere restriction fragment analysis

Telomere restriction fragment (TRF) analysis was performed with the TeloTAGGG Telomere Length Assay kit from Roche according to manufacturer’s instructions.

### C-circles

Detection of C-circles was performed as described in Henson *et al.* (30), using 30 ng of *Hinf*I/*Rsa*I-digested genomic DNA as template. Amplification products were deposited on a Hybond N+ nylon membrane (GE Healthcare) and hybridized with a radioactive (CCCTAA)_4_ probe labeled with polynucleotide kinase.

### RNA extraction, cDNA and quantitative reverse transcriptase-polymerase chain reaction

RNA extraction, cDNA synthesis and quantitative polymerase chain reaction (qPCR) were performed as described previously (12) using primers listed in Supplementary Table S2.

### Hirt procedure

Extrachromosomal DNA was isolated using a Hirt-extraction procedure as described previously (31). Briefly, 4 × 10^7^ cells were collected, washed twice with phosphate buffered saline, resuspended in 1 ml of buffer containing 10 mM Tris (pH 7.6), 10 mM EDTA and 0.6% sodium dodecyl sulfate, and incubated at room temperature (RT) for 15 min. After addition of 125 mM NaCl, solution was mixed gently and incubated overnight. Samples were centrifuged at 17 000 × *g* for 40 min at 4°C. Supernatant was collected as the low molecular weight (MW) DNA fraction and pellet contained the high MW DNA. DNA from both fractions was recovered by phenol-chloroform extraction followed by ethanol precipitation and samples were resuspended in 150 µl of 10 mM Tris (pH 7.6). DNA was separated on 0.8% agarose gel, transferred onto nylon membrane and hybridized with the telomeric probe provided in the TeloTAGGG Telomere Length Assay kit (Roche). After exposure on film, signals were quantified with Image J software.

### Micrococcal nuclease digestion-based analysis of chromatin condensation

Nuclei isolated from 10^7^ cells were digested with 4 U of micrococcal nuclease (MNase; Sigma)/mg DNA at 37°C for the indicated times (32). DNA was purified, separated on 1.5% agarose gel, transferred onto nylon membrane and hybridized with either the telomeric probe (TeloTAGGG Telomere Length Assay kit, Roche) or the control pBamX5 centromeric alphoid sequence probe (12) labeled with Megaprime DNA Labeling System (GE Healthcare) and [α^32^P]dCTP (PerkinElmer).

### TERRA blot

TERRA blots were performed as described previously (12).

### TERRA stability

Measurement of TERRA stability was performed as described previously, using Actinomycin D treatments (12).

### ChIP

ChIP was performed using standard procedures and antibodies listed in Supplementary Table S1. A detailed protocol is provided in Supplementary Methods. Purified DNA recovered by ChIP was denatured in 0.2 M NaOH by heating to 100°C for 10 min and spotted onto a positively charged Biodyne B nylon membrane (Pall, VWR) before hybridization with a digoxigenin-labeled telomeric C-rich oligonucleotide (CCCTAA)_4_TTA prepared using 3′-end labeling kit (Roche). Signal was revealed using the anti-digoxigenin-alkaline phosphatase antibodies (Roche) and CDP-Star (Roche), and images were captured using the Luminescent image analyzer LAS-4000 mini (GE Healthcare). Centromeric probe with sequence: 5′-CTTCGTTGGAAACGGGA (forward strand of the CenP Box) was used as a control. The primer was polyacrylamide gel electrophoresis purified and end-labeled with [γ^32^P]dATP (PerkinElmer) using PNK followed by purification on G-25 column.

## RESULTS

### Comparison of ALT and TEL+ IMR90-derived fibroblasts reveals distinct chromatin features at telomeric repeats

**Figure 1.**
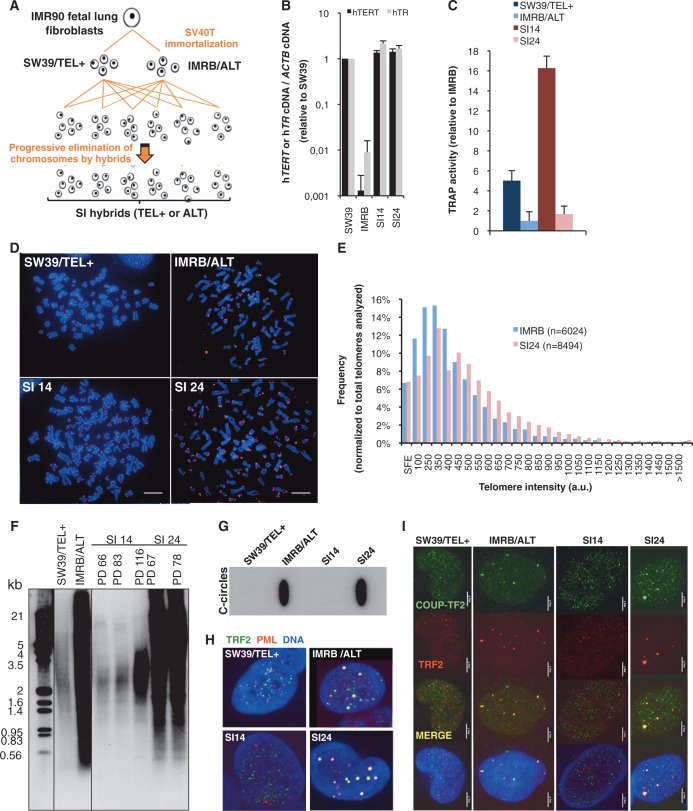
Characterization of TMM in cellular hybrids between SW39/TEL+ and IMRB/ALT. (**A**) Overview of hybridization procedure. Parental cells were fused with PEG and hybrids were selected in the presence of antibiotics before cloning. (**B**) Expression levels of hTERT (black bars) and hTR (gray bars) telomerase subunits in SW39/TEL+ and IMRB/ALT parental cell lines and in SI14 and SI24 hybrids. Values were first normalized to β-actin (ACTB) and then to SW39/TEL+; s.d. are shown. (**C**) Telomerase activity assayed by qTRAP in SW39/TEL+, IMRB/ALT, SI14 and SI24 cell lines. TRAP activity was normalized to IMRB/ALT; standard deviations (s.d.) are shown. (**D**) Telomeric FISH using a telomeric Cy3-(TTACCC)_3_ PNA probe on metaphase spreads. DNA is stained with DAPI (blue). (**E**) Distribution of telomere length in IMRB/ALT (blue bars) and SI24 (pink bars) cells. Telomere length was evaluated by Q-FISH and given an arbitrary value based on fluorescence intensity. The total number of telomeres analyzed in both cell lines is indicated (*n*). SFE: signal-free ends. (**F**) Terminal Restriction Fragment analysis of telomeres in parental cell lines and in SI14 and SI24 hybrids clones at various population doubling (PD) values. Molecular weight markers are shown (kb). (**G**) C-circles in SW39/TEL+, IMRB/ALT, SI14 and SI24 detected by the assay described previously (30). (**H**) Detection of APBs by immunofluorescence using antibodies against TRF2 (green) and PML (red). (**I**) Detection of COUP-TF2 orphan receptor binding at telomeres by immunofluorescence using antibodies against COUP-TF2 (green) and TRF2 (red).

To compare chromatin properties at telomeres of ALT and TEL+ cells, we wished to avoid working with cells of too divergent genetic backgrounds. To this end, we chose two cell lines derived from SV40-immortalization of IMR90 fetal lung fibroblasts: IMRB, a cell line that maintains telomeres through an ALT mechanism, and SW39, which relies on telomerase (28). To increase the number of genetically comparable ALT and TEL+ cell lines, we generated cellular hybrids between SW39/TEL+ and IMRB/ALT (Figure 1A). Freshly isolated hybrids randomly eliminate a subset of parental chromosomes before stabilizing their karyotype and eventually display either the ALT or the TEL+ phenotype (33). After initial characterization of obtained clones, two clones (SI14 and SI24), were selected for further experiments. These two clones express markers from each parent and both hTERT and hTR telomerase subunits (Supplementary Figure S1 and Figure 1B). However, while telomerase activity is detectable in SI14, it is not in the SI24 clone. Furthermore, telomeric FISH revealed a homogeneous profile, characteristic of TEL+ cells, for SI14 and a heterogeneous profile, reminiscent to that of IMRB/ALT parent, for SI24 (Figure 1C and D). Importantly, the frequency of signal-free ends that we measured by Q-FISH was similar in IMRB (6.7%) and SI24 (6.8%), indicating that telomerase is not active in these cells (Figure 1E). Telomere heterogeneity of SI24 was also observed by TRF analysis (Figure 1F). The ALT phenotype of SI24 was confirmed by the presence of (i) extrachromosomal telomeric repeat (ECTR) species, among which C-circles are readily detectable (30) (Figure 1G), and (ii) APBs, revealed by the colocalization of TRF2 and PML proteins (Figure 1H). Furthermore, agreeing with recent reports of orphan receptor binding at ALT telomeres (4,5), we showed the presence of COUP-TF2 at telomeres of both IMRB and SI24 ALT cell lines (Figure 1I). None of these ALT hallmarks was detected in SI14, firmly establishing the TEL+ phenotype of SI14 hybrid.

**Figure 2.**
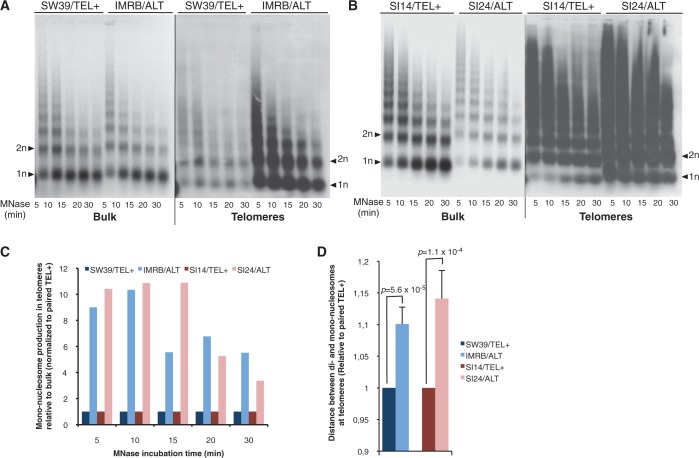
Telomeric chromatin is less condensed in ALT than in TEL+ cells. (**A**) MNase digestion assays in SW39/TEL+ and IMRB/ALT cells. Digestion patterns of bulk chromatin were visualized with ethidium bromide staining (Bulk); telomeric chromatin patterns (Telomeres) were observed by Southern blot with a telomeric probe. Mono (1n)- and di (2n)-nucleosomes are shown by arrowheads. Time of digestion with MNase (min) is indicated below. (**B**) Same as (A) for SI14/TEL+ and SI24/ALT cell lines. (**C**) Quantifications from (A) and (B). Ratios of mono-nucleosomes over total signals, for either bulk chromatin or TTAGGG repeats, were calculated for the various incubation times with MNase. Ratios obtained for telomeric chromatin were then normalized to the corresponding ratios measured for bulk chromatin. Finally, relative ratios were normalized to their respective TEL+ counterparts. (**D**) Relative distances between di- and mono-nucleosomes at telomeres of ALT cells compared with their respective TEL+ counterpart. Distances were normalized to either SW39/TEL+ or SI14/TEL+. Standard deviations (s.d.) are shown and *P* values were calculated using Student *t* test.

To characterize telomeric chromatin, we first relied on the MNase digestion assay. Ethidium bromide staining of bulk chromatin revealed similar ability of MNase to digest chromatin into mono-nucleosomes in SW39/TEL+ and IMRB/ALT cells, suggesting that, at the bulk level, chromatin condensation is similar in those two cell lines (Figure 2A). However, accessibility of telomeric chromatin to MNase digestion was clearly distinct between ALT and TEL+ cells, as shown by Southern blot analysis with a telomeric probe. Although mono-nucleosomes were readily detected on digestion of telomeric chromatin in IMRB/ALT cells, accessibility of MNase to telomeres of SW39/TEL+ cells was reduced (Figure 2A and C and Supplementary Figure S2A and B). Consistently, MNase assays in SI14/TEL+ and SI24/ALT cells revealed differences in the high-order structure of telomeres, with a more open chromatin at SI24/ALT telomeres (Figure 2B and C). The increased accessibility to MNase digestion that we uncovered at telomeres of ALT cells compared with TEL+ was not observed at the level of α-satellite centromeric repeats, suggesting that differences in chromatin compaction may be restricted to telomeres (Supplementary Figure S2C–F).

**Figure 3.**
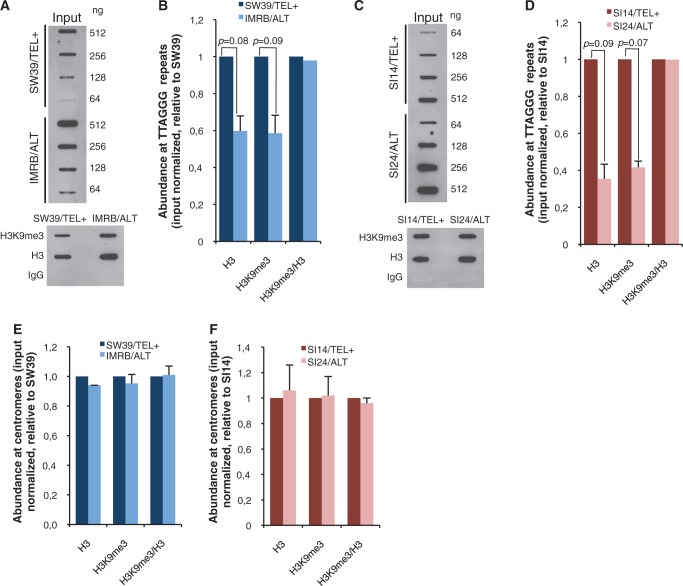
Reduced nucleosome density at ALT telomeres. (**A**) ChIP against H3 and H3K9me3 at telomeres of SW39/TEL+ and IMRB/ALT cells. *Upper panel*, serial dilutions of input chromatin samples. Amounts (ng) of blotted chromatin are indicated on the right. *Lower panel*, representative ChIP experiments. Control ChIP experiments with IgG are shown. Telomeric DNA was detected using a C-rich telomeric probe. (**B**) Quantifications from (A). ChIP signals in SW39/TEL+ (dark blue) or IMRB/ALT (light blue) were first normalized to telomeric DNA input and then to SW39/TEL+ (*n* = 3 independent experiments); standard deviations (s.d.) are shown and *P* values were calculated using Student *t* test. H3K9me3/H3 ratios were normalized to SW39/TEL+. (**C** and **D**) Same as (A) and (B) for SI14/TEL+ (purple) and SI24/ALT (pink) cell lines. (**E** and **F**) Relative H3 and H3K9me3 densities at centromeres in SW39/TEL+ (dark blue), IMRB/ALT (light blue), SI14/TEL+ (purple) and SI24/ALT (pink) cell lines. ChIP signals were first normalized to centromeric DNA input and then to their respective TEL+ counterpart. Representative blots used for quantification are shown in Supplementary Figure S4A and B.

We also noticed that the spacing between the signals obtained for mono- and di-nucleosomes was higher at ALT than at TEL+ telomeres, suggesting that the average nucleosome repeat size was bigger at ALT telomeres, whether in the SW39/IMRB or in the SI14/SI24 pair of TEL+/ALT cell lines (Figure 2A, B and D). These data suggested that ALT telomeres may be characterized by a lower nucleosome density than TEL+ telomeres. To investigate this further, we performed ChIP experiments using antibodies against H3 or H4 core histones. In agreement with MNase assays, ChIP results revealed reduced density of about two times for both H3 (Figure 3A–D) and H4 (Supplementary Figure S3A–C) at ALT telomeres. We next assessed H3K9me3 density and found that occupancy of H3K9me3 at ALT telomeres was reduced in the same proportion than telomeric H3 density (Figure 3A–D). Same conclusions were obtained for H4K20me3 telomeric density (Supplementary Figure S3A–C). Such differences in H3 or H3K9me3 densities were, however, not detected at the level of centromeric DNA (Figure 3E and F and Supplementary Figure S4A and B). Hence, compared with TEL+, telomeres from ALT cells are characterized by a drastic reduction of both H3 and H3K9me3 density, in perfect agreement with the telomeric chromatin decondensation uncovered by the MNase assay. However, ALT-dependent telomere maintenance does not affect telomeric H3K9me3/H3 ratios, as we obtained similar values in TEL+ and ALT cells, and this in both pairs of cell lines (Figure 3B and D).

To our knowledge, these data provide the first demonstration of differences in chromatin properties of ALT and TEL+ telomeres and are consistent with the hypothesis that a more open telomeric chromatin may favor ALT mechanism.

### Extrachromosomal telomeric DNA represents a minor fraction of bulk telomeric DNA repeats

**Figure 4.**
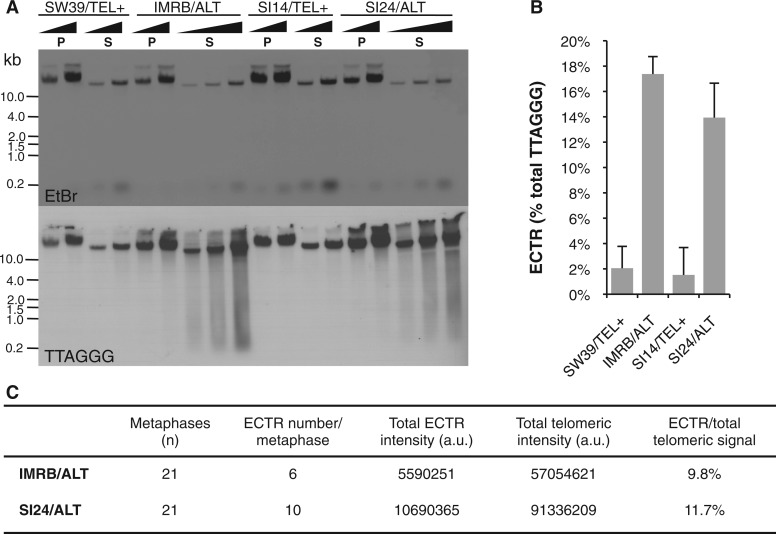
Quantification of ECTR in ALT cells. (**A**) Analysis of ECTR using Hirt procedure. Serial dilutions of pellets (P) or supernatants (S) obtained from SW39/TEL+, IMRB/ALT, SI14/TEL+ and SI24/ALT cell lines were analyzed by ethidium bromide staining (*upper panel*) or Southern blot to reveal telomeric repeats (*lower panel*). (**B**) Quantification of (A): ECTR abundance is depicted as percentage ± s.d. of total TTAGGG content obtained by dividing the signal coming from the smear between 0.2 and 10 kb in the S fraction by the signal coming from the sum of S and P fractions. (**C**) Analysis of ECTR using Q-FISH. Metaphase spreads were hybridized with a Cy3-O-O-(CCCTAA)_3_ PNA probe. Telomeric and ECTR intensities were evaluated by Q-FISH and given an arbitrary value based on fluorescence intensity. Representative pictures are shown in Supplementary Figure S5.

The above data strongly supported the presence of a more relaxed chromatin at ALT telomeres when compared to TEL+ cells. However, analyses of telomeric chromatin composition rely on the use of TTAGGG-containing probes that do not allow to distinguish between ECTRs and telomeres from ALT cells. ALT cells are indeed characterized by the presence of telomeric repeats that are not part of chromosomes and it is unknown whether chromatin properties of these ECTR species reflect those of ALT telomeres. In this context, it was thus important to evaluate the contribution of ECTR to the bulk of telomeric DNA repeats in both pairs of cell lines. To this end, we relied on a procedure described previously to separate high-molecular weight chromosomes (recovered in the pellet) from DNA species of lower molecular weight (in the supernatant) (31). Pellets and supernatants were analyzed separately after electrophoresis, blotting and hybridization with a telomeric probe (Figure 4A). Considering that the smears appearing exclusively in the supernatant fractions of ALT cells represent ECTR molecules, quantifications indicated that these species amount to 14–17% of total telomeric DNA repeats in IMRB/ALT and SI24/ALT cell lines (Figure 4B). Similar results were obtained by quantitative FISH, which revealed ECTR content of, respectively, ∼10 and 12% of total TTAGGG repeats in IMRB/ALT and SI24/ALT cell lines (Figure 4C and Supplementary Figure S5). Having established that ECTR contribute to 10–15% of total TTAGGG content of ALT cells, we considered that our MNase and ChIP analyses with telomeric probes mostly reflected chromatin status of telomeres.

### TERRA expression is higher in ALT than in TEL+ SV40T-immortalized IMR90 fibroblasts

We showed recently that TERRA production is inversely correlated with telomeric H3K9me3 density at human telomeres and that telomere transcription can be repressed by increased density of telomeric histone marks (12). In light of recent evidences pointing toward a positive impact of telomeric RNA on HR at yeast telomeres (26), and since we found that H3K9me3 density is lower at ALT than at TEL+ telomeres, we wished to test whether telomere transcription may be upregulated in ALT cells.

**Figure 5.**
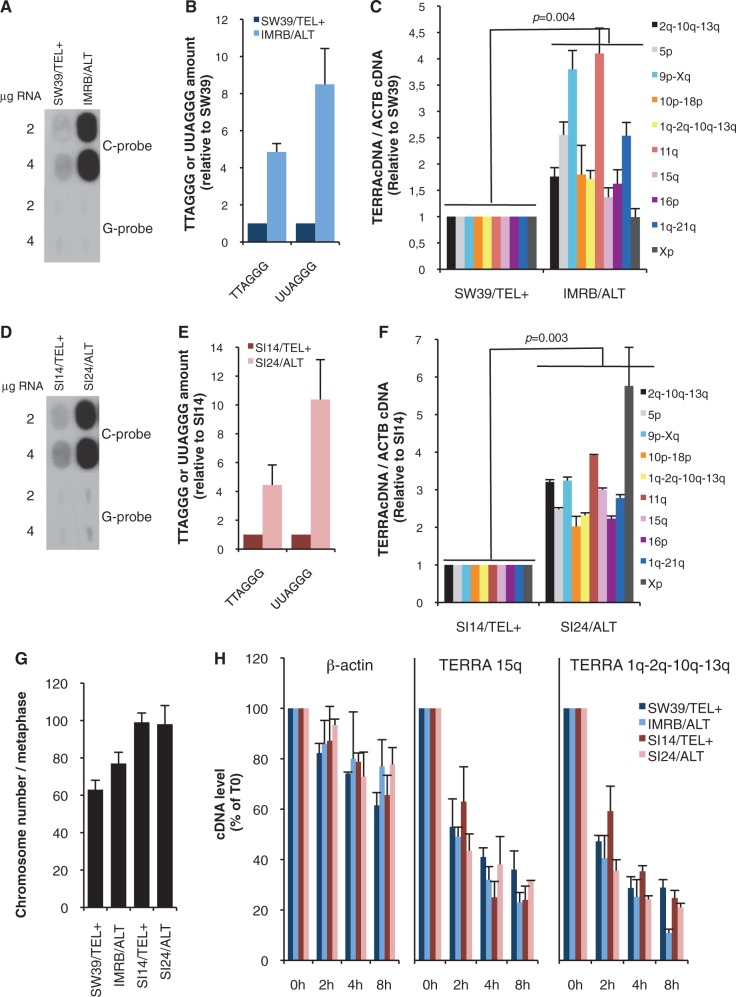
TERRA expression is upregulated in ALT compared with TEL+ cells. (**A**) RNA-blot to quantify UUAGGG content in SW39/TEL+ and IMRB/ALT cell lines. Two or 4 µg of total RNA was analyzed with either a C-rich or a G-rich (negative control) probe. (**B**) Quantifications of (A) and of Figure 3A in SW39/TEL+ (dark blue) and IMRB/ALT (light blue), normalized to SW39/TEL+ (*n* = 3); s.d. are shown. (**C**) qRT-PCR analyses of various TERRA molecules (indicated on the right), normalized first to β-actin cDNA and then to SW39/TEL+. Three independent RT were performed, s.d. are shown and *P* values were calculated using Student *t* test. (**D–F**) Same as (A–C) for SI14/TEL+ and SI24/ALT cell lines. Quantifications of TTAGGG repeats in SI14/TEL+ and SI24/ALT cells were based on Figure 3C. (**G**) Number of chromosomes in SW39/TEL+, IMRB/ALT, SI14/TEL+ and SI24/ALT cell lines evaluated by manual counting on metaphase spreads. At least 21 metaphases were analyzed in each case. (**H**) TERRA stability was evaluated by qRT-PCR after treatment of SW39/TEL+ (dark blue), IMRB/ALT (light blue), SI14/TEL+ (purple) or SI24/ALT (pink) cell lines with Actinomycin D for the indicated times. Primers amplifying either 15q- or 1q/2q/10q/13q-derived TERRA molecules were used. Stability of β-actin mRNA was monitored as control. In all cases, values were normalized to 0 h time point. *n* = 3; s.d. are shown.

Importantly, since telomere transcription is regulated by subtelomeric promoter methylation (34), we first checked that TERRA promoters displayed similar DNA methylation levels in SW39/TEL+ and IMRB/ALT cells using bisulfite sequencing (Supplementary Figure S6). Next, we measured TERRA abundance. As reported previously, each TERRA molecule comprises a chromosome-specific 5′ end followed by a tract of UUAGGG repeats, the length of which is proportional to telomere length (12). For a first quantification of TERRA amounts in TEL+ and ALT cells, we measured, on one hand, the total amounts of UUAGGG repeats and, on the other hand, the total levels of TTAGGG repeats in SW39/TEL+ and IMRB/ALT cell lines (Figure 5A and B). In agreement with our previous observations that TERRA length correlates with telomere length (12,35), we found that increased TTAGGG levels were associated with higher UUAGGG content in IMRB/ALT cells. However, UUAGGG/TTAGGG ratio was higher in IMRB/ALT than in SW39/TEL+ cells, suggesting that transcription of ALT telomeres may be upregulated. Accordingly, quantitative reverse transcriptase-PCR (qRT-PCR) analyses of individual TERRA molecules, using primers that recognize TERRA 5′ ends, revealed significantly higher levels of TERRA in IMRB/ALT cell line when compared with SW39/TEL+ (*P* = 0.004, Figure 5C). Upregulation of TERRA expression was not homogeneous between chromosome ends and varied between ∼1- and 4.5-fold (Figure 5C). Consistent results were obtained by measuring TERRA levels in SI14/TEL+ and SI24/ALT cell lines (Figure 5D–F). Importantly, we checked, by counting chromosome numbers on metaphase spreads, that the differences in TERRA levels that we detected between SW39/TEL+ and IMRB/ALT on one hand, and between SI14/TEL+ and SI24/ALT, on the other hand, did not result from highly divergent chromosome numbers within these two pairs of cell lines (Figure 5G). Higher levels of TERRA molecules in ALT cells may result either from increased telomere transcription or from increased TERRA stability. To discriminate between these two possibilities, we treated cells with Actinomycin D RNA PolI/II inhibitor and quantified TERRA molecules by qRT-PCR in a time-course experiment. Our results revealed similar stability of TERRA molecules regardless of the TMM (Figure 5H).

The above data revealed an upregulation of TERRA in ALT compared with TEL+ cells and confirmed our previous observations that, in comparable genetic backgrounds, lower levels of telomeric H3K9me3 density are associated with increased TERRA production.

### Telomerase-mediated elongation of short ALT telomeres increases telomeric chromatin condensation and represses transcription

**Figure 6.**
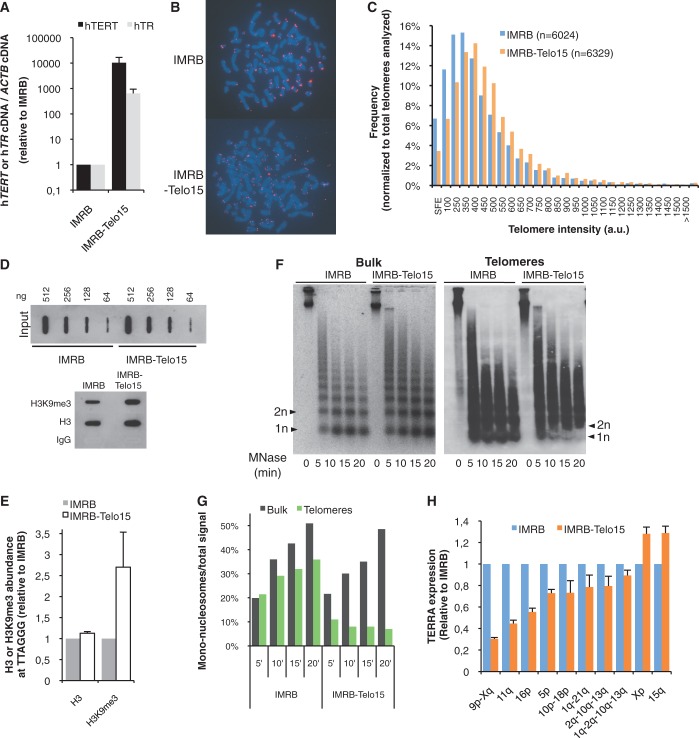
Telomerase-mediated elongation of short ALT telomeres increases chromatin condensation and represses transcription. (**A**) Overexpression of hTERT and hTR subunits in IMRB cells. hTERT (black bars) and hTR (gray bars) cDNA levels were quantified by qRT-PCR in IMRB and IMRB-Telo15 cells. Values were normalized first to ACTB cDNA levels and then to IMRB. (**B**) Representative pictures of FISH with a telomeric probe (red) in IMRB and IMRB-Telo15 cells. DNA is stained with DAPI (blue). (**C**) Distribution of telomere length in IMRB (blue bars) and IMRB-Telo15 (orange bars) cells. Telomere length was evaluated by Q-FISH and given an arbitrary value based on the fluorescence intensity. The total number of telomeres analyzed in both cell lines is indicated (*n*). SFE: signal-free ends. (**D** and **E**) Quantification of H3 and H3K9me3 density at telomeres of IMRB and IMRB-Telo15 by ChIP. See Figure 3 legend for details. (**F** and **G**) MNase digestion assays in IMRB and IMRB-Telo15 cells as described in Figure 2 legend. (**H**) qRT-PCR analyses of TERRA molecules in IMRB and IMRB-Telo15 cells, normalized first to ACTB cDNA and then to IMRB (*n* = 3); s.d. are shown.

Our results indicated that ALT telomeres are less condensed than TEL+ telomeres and are more transcribed. However, a drawback associated with the use of telomeric probes in MNase and ChIP experiments is that, because of the presence of some long telomeres in ALT cells, the numerous short telomeres only poorly contribute to the pool of TTAGGG repeats analyzed. Thus, these chromatin analyses of bulk TTAGGG repeats do not give valuable information about the chromatin status of short ALT telomeres. Because we concluded from our experiments that reduced density of telomeric H3K9me3 marks at bulk TTAGGG sequences correlates with increased transcription, we wished to investigate whether short telomeres of IMRB/ALT cells nevertheless contribute to the overall increased TERRA expression in these cells. Because of the above-mentioned problems to properly evaluate chromatin status at short ALT telomeres, we reasoned that, if H3K9me3 density was similarly low at short telomeres, artificially increasing HC marks of these short telomeres would repress their transcription. To do this, we overexpressed telomerase based on (i) previous reports establishing that telomerase overexpression is able to elongate the short ALT telomeres (36,37) and (ii) our previous data that telomerase-mediated lengthening of telomeres increases H3K9me3 density (12). As expected, short telomeres of IMRB cells were elongated on overexpression of both telomerase subunits, hTERT and hTR, in IMRB-Telo15 cells (Figure 6A–C) and, in agreement with our previous study (12), telomeric H3K9me3 density was increased (Figure 6D and E). Fitting with our previous hypothesis that telomere elongation increases H3K9me3 density through a possible upregulation of histone methyltransferase recruitment, rather than by increasing global H3 density, telomeric H3 density was only slightly increased in IMRB-Telo15 cells (Figure 6D and E). Agreeing with a rise in H3K9me3 density at telomeres of IMRB-Telo15 cells, we found that MNase access to telomeres was reduced (Figure 6F and G). This increase in H3K9me3 density appeared to be specific to telomeres and was not detected at centromeric regions of IMRB-Telo15 cells (Supplementary Figure S7A and B). Accordingly, IMRB-Telo15 cells displayed reduced expression levels of TERRA molecules produced from a subset of chromosome ends (Figure 6H). Similar results were obtained with another IMRB-Telo7 clone that we isolated (Supplementary Figure S8A–D). Altogether, we concluded that short ALT telomeres contribute to global increased telomeric transcription in ALT cells, most likely because their chromatin is also more open.

### Downregulation of ATRX does not increase TERRA levels

The results from this study strongly suggest that reduced H3K9me3 density at ALT telomeres was responsible for increased telomere transcription rate. However, recent studies indicated that ALT phenotype is frequently characterized by the loss of ATRX (16–18), a chromatin remodeler that has been associated with repression of TERRA in mouse embryonic stem (ES) cells (38). We thus wished to investigate whether the upregulation of ALT telomere transcription that we detected in paired ALT/TEL+ cells may be a direct consequence of ATRX deficiency.

**Figure 7.**
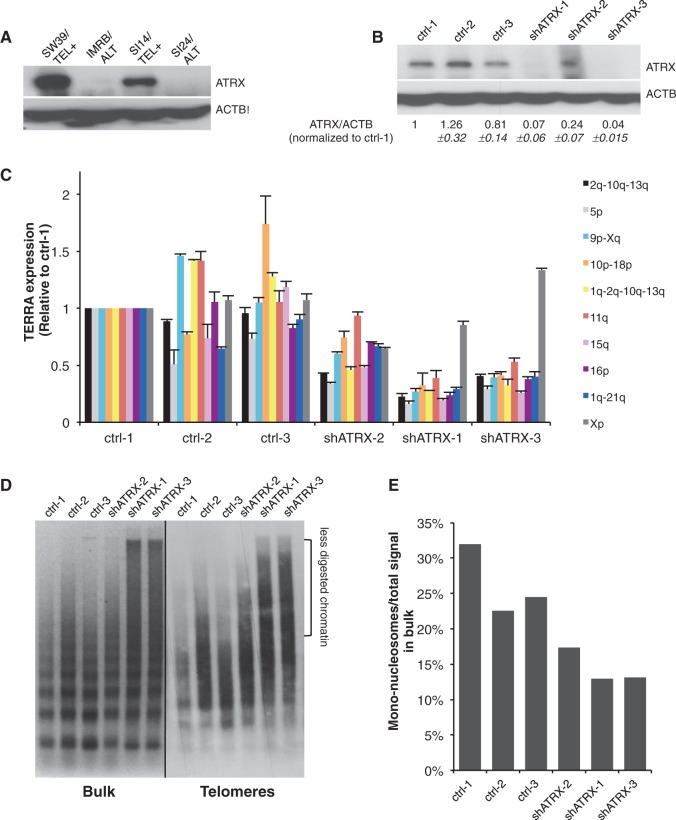
ATRX loss does not increase TERRA expression. (**A**) Western blot analysis of ATRX in SW39/TEL+, IMRB/ALT, SI14/TEL+ and SI24/ALT. β-actin is shown as loading control. (**B**) Western blot analysis of ATRX in SW39 shATRX, clones 1–3, compared with SW39 control clones 1-3. ATRX protein levels, normalized first to β-actin, and then to SW39 ctrl-1, are indicated below. (**C**) qRT-PCR analyses of various TERRA molecules (indicated on the right) in SW39 shATRX clones 1–3 and control clones, normalized first to β-actin cDNA and then to SW39 ctrl-1 (*n* = 3). (**D**) MNase digestion assays in SW39 control clones 1–3 and SW39 shATRX clones 1–3 as described in Figure 2 legend and after 10 min of digestion. (**E**) Quantification of (D). Similar results were obtained for other digestion times (data not shown).

First, western blot analyses confirmed the loss of ATRX expression in IMRB/ALT and SI24/ALT cell lines (Figure 7A), and cDNA sequencing revealed the presence of a premature STOP codon at position 1730 in exon 9. Conversely, ATRX protein was detected in both SW39/TEL+ and SI14/TEL+ cells (Figure 7A). Next, to test the impact of ATRX loss on TERRA expression in IMR90-derived fibroblasts, we knocked-down ATRX with an shRNA construct in SW39/TEL+ cell line (Figure 7B). In contrast to what has been reported in mouse ES cells, ATRX knockdown did not upregulate TERRA expression but reduced TERRA levels (Figure 7C). We checked that the reduction in TERRA expression was not due to telomere elongation (Supplementary Figure S9). Agreeing with reduced telomeric transcription, we found that chromatin digestion by MNase is strongly impaired on ATRX knockdown, both at the level of bulk chromatin and at telomeres, suggesting that chromatin condensation is increased in these cells (Figure 7D and E).

These data therefore suggested that loss of ATRX is unlikely to be directly responsible for increased TERRA expression or chromatin decondensation in ALT cells.

## DISCUSSION

ALT cells are characterized by the presence of heterogeneous telomeres, ranging from very short to very long. In such cells, telomeric repeats are not synthesized by telomerase but are maintained through homologous recombination processes and are characterized by a high abundance of repeat variants that are distinct from the canonical TTAGGG repeats and act as binding sites for orphan receptors (4,5). Recombination events between telomeric DNA sequences are normally repressed at telomeres but these regulatory mechanisms appear to be lost in ALT cells. The reasons for derepression of HR at ALT telomeres are not elucidated yet but experiments in mouse knockout mutants affected in HC formation suggested that downregulation of telomeric HC marks may contribute to ALT phenotype (13). Chromatin structure has indeed long been regarded as a strong candidate for a determinant of homologous recombination. Multiple evidences in yeast point toward an important role for nucleosome depletion in HR activity at meiotic recombination hotspots (39). Similarly, nucleosome profiling at four mouse recombination hotspots confirmed the presence of largely open chromatin structures (40). More recently, *in vivo* and *in vitro* studies performed in *Saccharomyces cerevisiae* went deeper into the understanding of the relationship between HR and chromatin composition and showed that DNA end resection, the first step in HR pathways, is inhibited by the presence of nucleosomes (41). Posttranslational histone H3 modifications, like acetylation of lysine 9 or trimethylation of lysine 4, also appear to be strong determinants of HR efficiency (42)and acetylation of H4K5/K12 was recently reported to facilitate recruitment of key HR protein RAD51 at damaged chromatin (43).

### Chromatin is decondensed at ALT telomeres

**Figure 8.**
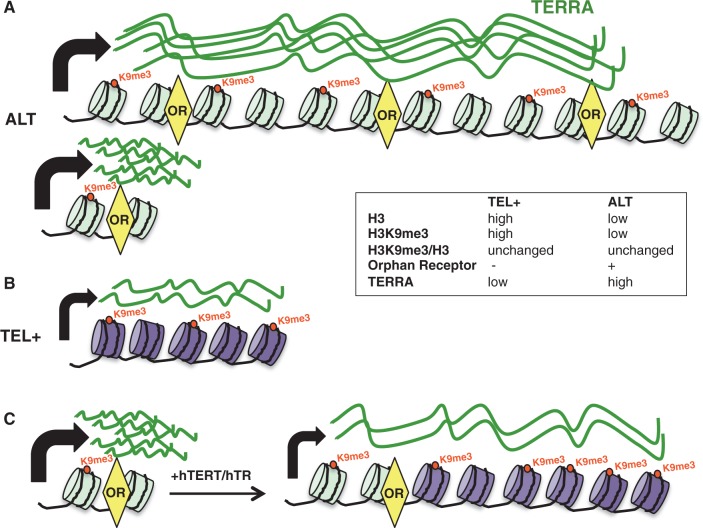
Possible organization of chromatin at telomeres of ALT and TEL+ cells. (**A**) ALT telomeres are heterogeneous in size, ranging from very long to very short telomeres. They are characterized by a lower nucleosome density than the one detected at telomeres of comparable TEL+ cells. Reduced nucleosome density at ALT telomeres correlates with decreased H3K9me3 occupancy and increased transcription rate, whether at long or at short telomeres. Because of the presence of repeat variants, ALT telomeres are also bound by orphan receptor proteins that, as we speculate, may play important roles in the HR-dependent mechanism of telomere maintenance. (**B**) Telomeric chromatin of TEL+ cells is more condensed; H3 and H3K9me3 densities are increased and transcription is decreased. (**C**) On overexpression of h*TERT* and h*TR* in ALT cells, short telomeres are elongated. This results in increased H3K9me3 density and telomere transcription downregulation. OR: orphan receptor. H3K9me3 groups are indicated by red dots and TERRA molecules are shown in green.

Understanding the defects that underlie telomeric recombination deregulation in human cells is undoubtedly an important challenge in the context of anti-cancer therapies targeting telomeres (6). Here, we analyzed two pairs of cell lines derived from IMR90 human fetal lung fibroblasts to investigate, in similar cellular backgrounds, the chromatin structure of ALT and TEL+ telomeres. Fitting with the proposal that lower levels of chromatin condensation may promote telomeric recombination, MNase assays revealed reduced chromatin compaction at ALT telomeres and ChIP analyses confirmed the lower occupancy of both H3 and H3K9me3 (Figure 8A and B). Based on our results, we speculate that telomeric chromatin decondensation might be one of the driving events of ALT occurrence.

How can we explain the reduced nucleosome density of ALT telomeres? Recently, exome sequencing studies performed in a large number of tumors identified recurrent ATRX mutations in ALT tumors (16–18) and we similarly found that ATRX protein is neither expressed in IMRB/ALT nor in SI24/ALT cells. However, whether and how ATRX loss may drive telomeric chromatin remodeling during the process of ALT activation is still unknown, as ATRX depletion alone is not sufficient to promote telomeric recombinations (18). It was, however, reported that ATRX loss may facilitate ALT establishment during *in vitro* immortalization of human fibroblasts with SV40T antigen (33), suggesting that ATRX loss may impact on telomeric HC when this happens during the telomere shortening phase that precedes activation of a TMM. Hence, it is possible that ATRX loss may facilitate subtelomeric DNA invasion by shortened telomeres. In this scenario, subtelomeric DNA invasion would be followed by repeat variant acquisition and recruitment of orphan receptors at telomeres that, in turn, may be involved in chromatin remodeling. Supporting this hypothesis, previous studies described an interaction of nuclear receptors with multiple chromatin remodeling complexes (44,45) and a recent work reported that the recruitment of COUP-TF1/2 orphan receptors to their binding sites is associated with low-nucleosome occupancy and active transcription (46). Thus, based on these evidences, we speculate that recruitment of orphan receptors to ALT telomeres may alter their heterochromatic state. Agreeing with this, we showed that telomerase-dependent elongation of short ALT telomeres, by adding TTAGGG repeats which are not bound by orphan receptors, increases telomeric chromatin compaction even though the cellular context is still of the ALT type (Figure 8C).

### TERRA expression is higher in ALT than in TEL+ cells

In agreement with our previous study that correlated increased occupancy of H3K9me3 at telomeres with repression of transcription (12), our data indicated that, when compared to TEL+ counterparts, transcription is upregulated at ALT telomeres. These data fit with a previous report that SV40-immortalized ALT cells have higher levels of TERRA than comparable TEL+ cells (47). In that study however, DNA hypomethylation of subtelomeres was partly accounting for the increased TERRA levels and the possible impact of telomeric chromatin was not evaluated (47). Although we detected a good correlation between low telomeric H3K9me3 density and high TERRA expression, we had to consider the possibility that elevated rate of ALT telomere transcription may result from ATRX loss as mouse ES cells derived from ATRX knockout animals upregulate TERRA expression (38). However, arguing against this possibility, we detected lower amounts of TERRA molecules in SW39/TEL+ fibroblasts expressing an shRNA construct against ATRX. The reasons for the discrepancies between mouse ES cells and human fibroblasts are not clear yet but our observations fit with the proposed role of ATRX as an activator of gene transcription that inhibits the deposition of macroH2A.1, an histone variant associated with transcriptionally inactive chromatin (48). Supporting this, we found that chromatin condensation is globally increased upon ATRX knockdown. On the other hand, we speculate that the recruitment of orphan receptors may promote telomere transcription. First, as suggested above, these receptors may participate in telomeric chromatin decondensation. Second, COUP transcription factors have been shown to activate a series of gene promoters, both *in vitro* and *in vivo* (49,50), and ChIP experiments showed that the recruitment of COUP-TF1 to target gene promoters is associated with active chromatin marked by H3K9 acetylation (51).

### Cross talk between telomere length and HC marks appears to be disrupted at ALT telomeres

Unlike the situation observed at elongated TEL+ telomeres (12), the overall increased abundance of TTAGGG repeats in ALT cells was not associated with increased density of H3K9me3 marks at telomeres as H3K9me3/H3 ratios were similar in paired TEL+ and ALT cells. Based on data obtained by Deng *et al.* (22), we previously proposed a model in which TERRA molecules transcribed from elongated telomeres are longer and may recruit more heterochromatinization factors, leading to increased telomeric H3K9me3 density (12). Our model further suggested the existence of a regulatory feedback mechanism aiming at repressing TERRA expression on telomere elongation as the rise in H3K9me3 marks observed in this case downregulates telomere transcription. This cross talk between telomere repeat abundance and H3K9me3 marks appears to be disrupted at ALT telomeres. How to explain then that H3K9me3 density at ALT telomeres is low despite the abundance of UUAGGG repeats? We propose several hypotheses: (i) because of the presence of telomeric repeat variants, TERRA sequences are altered and this may impact on the ability of RNA molecules to recruit heterochromatinization factors; (ii) the presence of orphan receptor molecules at ALT telomeres may interfere with the ability of TERRA molecules to induce heterochromatinization, as this process was reported to be mediated by TRF2/TERRA interactions (22) and orphan receptors compete with TRF2 for binding at telomeres (4). Our demonstration that telomerase overexpression in ALT cells appears to restore the correlation between telomere length and H3K9me3 density supports a role for telomerase-dependent telomeric repeat addition in TERRA-mediated heterochromatinization of telomeres, possibly linked to the sequence of telomeric repeats that are synthesized (TTAGGG instead of variants).

In summary, we showed that telomeric chromatin condensation is reduced at ALT compared with TEL+ telomeres. This is likely to facilitate HR-dependent telomere maintenance and to promote telomere transcription. In view of recent studies, it is tempting to speculate that upregulation of telomere transcription may as well contribute to ALT. This could be achieved through formation of telomeric RNA/DNA heteroduplexes that favor replication fork collapses and activate homologous recombination at telomeres (26) and/or could result from the impairment of replication fork progression by the transcriptional process itself (27). We propose that acquisition of telomere repeat variants may be an important event in ALT establishment by offering binding sites for orphan receptors, which, in turn, may contribute to both telomeric chromatin decondensation and transcription upregulation. In the future, it would be interesting to investigate whether increased transcriptional activity *per se* and/or the presence of TERRA at telomeres of ALT cells contributes to recombination events.

## SUPPLEMENTARY DATA

Supplementary Data are available at NAR Online.

## FUNDING

Fonds National de la Recherche Scientifique [PDR T.0025.13]. The Telomere and cancer laboratory is Labellisé Ligue. Funding for open access charge: Fonds National de la Recherche Scientifique.

*Conflict of interest statement*. None declared.

## Supplementary Material

Supplementary Data
