# Supplementary material for: Alternative Lengthening of Telomeres is characterized by reduced compaction of telomeric chromatin
Source: Nucleic Acids Res. 2014 Feb 5;42(7):4391–405. doi: 10.1093/nar/gku114 (PMC3985679; doi:10.1093/nar/gku114)
Supplement: Supplementary Data [file supp_42_7_4391__index.html]

Alternative Lengthening of Telomeres is characterized by reduced compaction of telomeric chromatin — Alternative Lengthening of Telomeres is characterized by reduced compaction of telomeric chromatin — Supplementary Data 

# Alternative Lengthening of Telomeres is characterized by reduced compaction of telomeric chromatin

## Supplementary Data

files

**Files in this Data Supplement:**

- Supplementary Data - pdf file
