# Supplementary material for: Alternative Lengthening of Telomeres is characterized by reduced compaction of telomeric chromatin
Source: Nucleic Acids Res. 2014 Feb 5;42(7):4391–405. doi: 10.1093/nar/gku114 (PMC3985679; doi:10.1093/nar/gku114)
Supplement: Supplementary Data [file supp_gku114_nar-03172-x-2013-File010.pdf]

**Supplementary information for Episkopou *et al.***

# Alternative Lengthening of Telomeres is characterized by reduced compaction of telomeric chromatin

## Supplementary methods

### Metaphase preparation and FISH

For metaphase preparation, cells were incubated for 1.5 h with 10 ng/ml colcemid (Gibco). A hypotonic shock was achieved in 0.075 M KCl at 37°C for 20 min. Cells were fixed in ethanol/acetic acid solution (3:1, v/v) and washed three times in this fixing reagent. Metaphase spreads were obtained by dropping suspensions of fixed cells onto clean superfrost slides. The Q-FISH procedure was carried out as described, using a Cy3-O-O-$(CCCTAA)_3$ PNA probe (Panagene) (29). Briefly, cells were fixed in 4% formaldehyde in PBS for 2 min, washed 3 × 5 min in 2× SSC, treated with pepsin (1 mg/ml in 0.05 M citric acid pH 2 solution) for 10 min at 37°C, post-fixed, washed, and dehydrated though an ethanol series. 40 µl of a hybridization mix containing 10 nM telomeric C-rich PNA probe $(CCCTAA)_3$ labeled with Cy3 (Panagene) in 70% formamide, 10 mM Tris pH 7.4, and 1% blocking reagent (Roche, 11096176001) was applied to each slide. Slides were co-denatured in the presence of the probe by heating to 80°C for 3 min. After a 3 h hybridization at room temperature, slides were washed 2 x 15 min in 70% formamide, 20 mM Tris pH 7.4, then 3 × 5 min in 50 mM Tris pH 7.4, 150 mM NaCl, 0.05% Tween-20, dehydrated in successive ethanol baths, air-dried and mounted in Vectashield with DAPI (Vector, H-1100). Images of labeled metaphase spreads were acquired using Zeiss Axioplan 2 imaging system. Telomeric signals were quantified using the iVision software (Chromaphor). Telomeric signals were segmented using the Segmentation tool including a manual correction to include individual telomeres at all metaphasic chromosome ends. Average pixel intensities were quantified and corrected for the average local background intensities of metaphase spreads. Statistical analyses were done using the Wilcoxon rank-sum test.

### ChIP

Cells were crosslinked directly in cell culture dishes by adding 1/10$^{th}$ of the medium volume of 1x crosslinking solution (0.1M NaCl, 1 mM EDTA, 0.5 mM EGTA, 0.05M Tris pH8 and 10% formaldehyde (Thermo Scientific, 28908)) for 10 min at 37°C. Reaction was stopped by adding glycine to a final concentration of 0.125 M for 5 min at room temperature. Nuclei were prepared from fixed and washed cells by homogenization in cell lysis buffer (5 mM PIPES pH 8, 85 mM KCl, 0.5% NP-40) and centrifugation at 1800 × *g* for 10 min. Finally, nuclei from two 300 cm$^2$ flasks were lyzed in 1 ml nuclei lysis buffer (50 mM Tris-HCl pH 8, 10 mM EDTA pH 8, 1% SDS) and lysates were sonicated for 30 min (30 sec on / 30 sec off) in a Diagenode water bath-sonicator at speed 5. Following a centrifugation at 14000 rpm for 10 min at 4°C, the cleared supernatants were snapfrozen in liquid nitrogen and stored at -80°C. Sonication efficiency was routinely monitored on DNA gel-electrophoresis by depositing 2.5 µg of purified DNA from sonicated chromatin to ensure that the bulk of DNA fragments had an average length of < 250 bp. Sonicated chromatin containing 20 µg DNA was diluted 10 times in ChIP dilution buffer (167 mM Tris-HCl pH 8, 16.7 mM NaCl, 1.2 mM EDTA pH 8, 1.1% Triton X-100, 0.01% SDS) and pre-cleared for 2 h, rotating at 4°C, with 20 µl blocked beads (Dynabeads Protein A beads (Invitrogen) incubated for 2 h with 5 mg/ml BSA) before the overnight incubation with 2 µg of antibody (listed in Table S1). Bound material was recovered after a 2 h incubation, rotating at 4°C,

with 30 µl blocked beads. The beads were washed, for 10 min in each of the following Wash buffers: Low Salt Buffer (20 mM Tris-HCl pH 8, 150 mM NaCl, 2 mM EDTA pH 8, 1% Triton X-100, 0.1% SDS), High Salt Buffer (20 mM Tris-HCl pH 8, 500 mM NaCl, 2 mM EDTA pH 8, 1% Triton X-100, 0.1% SDS), LiCl Buffer (10 mM Tris-HCl pH 8, 0.25 M LiCl, 1 mM EDTA pH 8, 1% NP-40, 1% Na Deoxycholate) and twice, 5 min each, in TE. ChIPed material was eluted by two 15 min incubations at room temperature with 225 µl elution buffer (0.1 M NaHCO3, 1% SDS). Chromatin was reverse-crosslinked by adding 20 µl of 5M NaCl and incubated over night at 65°C and DNA was submitted to RNase and proteinase K digestion and extracted by phenol-chloroform. Purified DNA recovered by ChIP was denatured in 0.2 M NaOH by heating to 100°C for 10 min and spotted onto a positively charged Biodyne B nylon membrane (Pall, VWR). Membranes were hybridized at 42°C in 6× SSC, 0.01% SDS with 20 pmol of digoxigenin-labeled telomeric C-rich oligonucleotide $(CCCTAA)_4TTA$ prepared using 3' end labeling kit (Roche 03353575910). Following hybridization washes (twice 5 min in 2× SSC, 0.01% SDS and once 2 min in 0.1× SSC, 0.01% SDS) the signal was revealed using the anti-digoxigenin-alkaline phosphatase antibodies (Roche 11093274910) and CDP-Star (Roche) following the manufacturer's instructions. Images were obtained using the Luminescent image analyzer LAS-4000 mini (GE Healthcare).

**DNA methylation analysis**

Methylation analyses of subtelomeric promoters from 1q, 2q, 8p, 9p, 10q, 13q, 15q, 21q, Xq and Yq were performed by sequencing of bisulfite-treated DNA as described previously (12).

## Supplementary Tables

**Table S1. Antibodies used in this study.**

| Target | Species | Ac I/II | Application | Dilution | Reference |
|---|---|---|---|---|---|
| **TRF2** | Mouse | I | IF | 1:500 | Imgenex IMG-124A |
| **PML** | Rabbit | I | IF | 1:200 | Abcam ab53773 |
| **COUP-TF2** | Rabbit | I | IF | 1:150 | Abcam ab50487 |
| **H3** | Rabbit | I | ChIP | | Abcam ab1791 |
| **H3K9me3** | Rabbit | I | ChIP | | Abcam ab8898 |
| **IgG** | Rabbit | I | ChIP | | Abcam ab37415 |
| **H4** | Rabbit | I | ChIP | | Millipore 04-858 |
| **H4K20me3** | Rabbit | I | ChIP | | Abcam ab9053 |
| **ATRX** | Rabbit | I | WB | 1:5000 | ImTec A301-045A |
| **β-actin** | Mouse | I | WB | 1:10000 | Sigma A5441 |
| **Mouse IgG (Alexa 546)** | Goat | II | IF | 1:400 | Invitrogen A11003 |
| **Rabbit IgG (Alexa 488)** | Donkey | II | IF | 1:400 | Invitrogen A21206 |
| **Rabbit IgG (HRP)** | Goat | II | WB | 1:10000 | Enzo ADI-SAB-300 |
| **Mouse IgG (HRP)** | Goat | II | WB | 1:2000 | Santa Cruz sc-2005 |

**Table S2. Primers used in this study.**

| Primer name | Target | Application | Sequence | Reference |
|---|---|---|---|---|
| **ß-actin** | ß-actin RNA | Reverse transcription | AGT CCG CCT AGA AGC ATT TG | (19) |
| **Telo** | UUAGGG repeats | Reverse transcription | CCC TAA CCC TAA CCC TAA CCC TAA CCC TAA | (19) |
| **hTERT-F** | hTERT cDNA | qRT-PCR | CGG AAG AGT GTC TGG AGC AA | (28) |
| **hTERT-R** | hTERT cDNA | qRT-PCR | GGA TGA AGC GGA GTC TGG A | (28) |
| **hTR-F** | hTR cDNA | qRT-PCR | TTT GTC TAA CCC TAA CTA ACT GAG AAG | (28) |
| **hTR-R** | hTR cDNA | qRT-PCR | TTG CTC TAG AAT GAA CGG TGG A | (28) |
| **ACT-F-NA** | ACTB cDNA | qRT-PCR | TGT ACG CCA ACA CAG TGC TG | This study |
| **CH016** | ACTB cDNA | qRT-PCR | GCT GGA AGG TGG ACA GCG A | (28) |
| **DAL1-F** | DAL1 cDNA | qRT-PCR | GTA GTG GTC CAT AAA GAG ACA GAG A | This study |
| **DAL1-R** | DAL1 cDNA | qRT-PCR | GAT ACA AGT CAG TTG GGT TAG AAG A | This study |
| **FN1-F** | FN1 cDNA | qRT-PCR | AGA CCC CAG GCT CCC ATC A | This study |

| | | | | |
|---|---|---|---|---|
| **FN1-R** | FN1 cDNA | qRT-PCR | GGG AGC ATC CAG TTT GGT TGT | This study |
| **1q-2q-10q-13q F** | TERRA | qRT-PCR | GAA TCC TGC GCA CCG AGA T | (12) |
| **1q-2q-10q-13q R** | TERRA | qRT-PCR | CTG CAC TTG AAC CCT GCA ATA C | (12) |
| **1q-21q F** | TERRA cDNA | qRT-PCR | TCT CGG TGC GCA GGA TTC AGA | (12) |
| **1q-21q R** | TERRA cDNA | qRT-PCR | GTC ACA GAC CAG TTA GAA TG | (12) |
| **2q-10q-13q F** | TERRA cDNA | qRT-PCR | GTC AGA GAC CAG TTA GAA CG | (12) |
| **2q-10q-13q R** | TERRA cDNA | qRT-PCR | GGT GCG CAG GAT TCA GAG AG | (12) |
| **5p F** | TERRA cDNA | qRT-PCR | GAG TGC ATT AGC ATA CAG GTG | (12) |
| **5p R** | TERRA cDNA | qRT-PCR | TCC TAA TGC ACA CGT AAC AC | (12) |
| **9p-Xq F** | TERRA cDNA | qRT-PCR | TTC CGC ACT GAA CCG CTC TAA | (12) |
| **9p-Xq R** | TERRA cDNA | qRT-PCR | GCA GCC ATG AAT AAT CAA GGT | (12) |
| **10p-18p F** | TERRA cDNA | qRT-PCR | CCT TCT AAC TGG ACT CTG AC | (12) |
| **10p-18p R** | TERRA cDNA | qRT-PCR | GCC ACA GCG ACG GTA AAT AA | (12) |
| **11q F** | TERRA cDNA | qRT-PCR | CTG ATT ATT CAG GGC TGC AAA | (12) |
| **11q R** | TERRA cDNA | qRT-PCR | GCC GCA TCG ACG GTG AAT AA | (12) |
| **15q F** | TERRA cDNA | qRT-PCR | CAG CGA GAT TCT CCC AAG CTA AG | (12) |
| **15q R** | TERRA cDNA | qRT-PCR | AAC CCT AAC CAC ATG AGC AAC G | (12) |
| **16p F** | TERRA cDNA | qRT-PCR | TGT GTT TCA ACG CTG CAA CTG | (12) |
| **16p R** | TERRA cDNA | qRT-PCR | AGT TAG AAC GGT TCA GTG TG | (12) |
| **Xp F** | TERRA cDNA | qRT-PCR | GCA AAG AGT GAA AGA ACG AAG CTT | (12) |
| **Xp R** | TERRA cDNA | qRT-PCR | CCC TCT GAA AGT GGA CCA ATC A | (12) |
| **1q-2q-10q-13q-21q Fint/ext** | TERRA promoter | Methylation analysis | GGT TTT TGA TTT TGA TTA TTT AG | (12) |
| **1q-2q-10q-13q-21q Rext** | TERRA promoter | Methylation analysis | TTC TCT TTA ACA CAC ACC C | (12) |
| **1q-2q-10q-13q-21q Rint** | TERRA promoter | Methylation analysis | TTC TCC TCT ACA CAA ACT TC | (12) |
| **8p-9p-15q-Xq Fext/int** | TERRA promoter | Methylation analysis | GTT TTA ATT GGT TTT TGA TTT TG | (12) |
| **8p-9p-15q-Xq Rext** | TERRA promoter | Methylation analysis | TTA TCC TCT ACA CAA ATT TC | (12) |
| **8p-9p-15q-Xq Rint** | TERRA promoter | Methylation analysis | ATT CTC CTC AAA TCA AAC C | (12) |

## Supplementary legends

**Figure S1. Gene expression markers from SW39/TEL+ and IMRB/ALT parental cell lines in SI14 and SI24 hybrids.** Relative expression levels of *FN1* and *DAL-1* genes in SW39/TEL+, IMRB/ALT, SI14 and SI24 cell lines. We previously identified these two genes as being differentially expressed between SW39/TEL+ and IMRB/ALT (data not shown) and these were thus selected as markers of the two parents. *FN1* or *DAL-1* cDNA values were normalized to *ACTB* cDNA and depicted as percentage ± s.d. of the expression levels in either SW39/TEL+ (*FN1*) or IMRB/ALT (*DAL-1*).

**Figure S2. Additional MNase digestion assays in SW39/TEL+ and IMRB/ALT cell lines and control experiments for α-satellite centromeric chromatin. (A)** Micrococcal nuclease (MNase) digestion assays in SW39/TEL+ and IMRB/ALT cells. Digestion pattern of bulk chromatin was visualized with ethidium bromide staining (Bulk); telomeric chromatin patterns (Telomeres) were observed by Southern blot with a telomeric probe. Mono (1n)- and di (2n)-nucleosomes are shown by arrowheads. The amount of MNase used (U/mg DNA) is indicated below. **(B)** Ratios of mono-nucleosomes over total signals (%), for either bulk chromatin or telomeres, were calculated for the various concentrations of MNase indicated below. **(C)** Patterns of α-satellite centromeric repeat chromatin after MNase digestion in SW39/TEL+ and IMRB/ALT cells observed by Southern blot. Mono (1n)- and di (2n)-nucleosomes are shown by arrowheads. Time of digestion with MNase (min) is indicated below**. (D)** Quantification of (C). Ratios of mono-nucleosomes over total signals (%) are indicated for the two cell lines and at various incubation times with MNase. **(E-F)** Same as (C-D) in SI14/TEL+ and SI24/ALT cells.

**Figure S3. Analysis of H4 and H4K20me3 density at telomeres of ALT and TEL+ cells. (A)** ChIP against various histone marks at telomeres of SW39/TEL+, IMRB/ALT, SI14/TEL+ and SI24/ALT cells. Serial dilutions of input chromatin samples are shown on both sides. Amounts (ng) of blotted chromatin are indicated. Telomeric DNA was detected using a C-rich telomeric probe. **(B)** Quantifications from (A). ChIP signals in SW39/TEL+ (dark blue) or IMRB/ALT (light blue) were first normalized to telomeric DNA input and then to SW39/TEL+. H4K20me3/H4 ratios were normalized to SW39/TEL+. **(C)** Same as (B) for SI14/TEL+ (purple) and SI24/ALT (pink) cell lines.

**Figure S4. H3 and H3K9me3 abundance at centromeres of SW39/TEL+, IMRB/ALT, SI14/TEL+ and SI24/ALT. (A)** ChIP against H3 or H3K9me3 at centromeres of SW39/TEL+ and IMRB/ALT cells. Serial dilutions of input chromatin samples are shown on the left. Amounts (ng) of blotted chromatin are indicated. Centromeric DNA was detected using a 5'-CTTCGTTGGAAACGGGA probe (forward strand of the CenP Box). **(B)** Same as (A) for SI14/TEL+ and SI24/ALT cell lines.

**Figure S5. Representative pictures for extrachromosomal telomeric repeat (ECTR) content determination by quantitative FISH.** Quantitative telomeric FISH using a telomeric C-rich PNA probe (red) was performed on metaphase spreads of IMRB/ALT and SI24/ALT. Chromosomes were

counterstained with DAPI (blue). ECTR are indicated (white arrowheads). Semi-automatic segmentation of 21 metaphases per condition was perform to distinguish between telomeric repeats at the ends of chromosomes (green), ECTR (white) and background (yellow). Quantification of telomere intensity was done semi-automatically. Telomere ends that were not segmented automatically due to weak signals were selected manually and marked with green diamonds. ECTR were defined as signals that are at least as large as telomeric signals but are not associated with DAPI staining. The large ECTR present in ALT metaphases were absent from Muntjac metaphases spread on the same slides (not shown).

**Figure S6. Subtelomeric promoters display similar levels of DNA methylation in SW39/TEL+ and IMRB/ALT cells.** Bisulfite analysis of subtelomeric promoter methylation using a combination of primers targeting various chromosome ends. The number of CpG sites analyzed is indicated above the bars and values are given as $^{me}$CpG/total CpG (%).

**Figure S7. H3 and H3K9me3 abundance at centromeres of IMRB and IMRB-Telo15 cells. (A)** ChIP against H3 or H3K9me3 at centromeres of IMRB and IMRB-Telo15 cells. Serial dilutions of input chromatin samples are shown in the above panel. Amounts (ng) of blotted chromatin are indicated. Centromeric DNA was detected using a 5'-CTTCGTTGGAAACGGGA probe (forward strand of the CenP Box). **(B)** Quantifications from (A). ChIP signals in IMRB and IMRB-Telo15 were first normalized to centromeric DNA input and then to IMRB. *n*=3 independent experiments; standard deviations are shown.

**Figure S8. Reduced accessibility of MNase to telomeres and down-regulation of TERRA expression in another IMRB-Telo clone. (A)** Overexpression of hTERT and hTR subunits in IMRB cells. hTERT (black bars) and hTR (grey bars) cDNA levels were quantified by qRT-PCR in IMRB, IMRB-Telo15 and IMRB-Telo7 cells. Values were normalized first to ACTB cDNA levels and then to IMRB. **(B)** MNase digestion assays in IMRB, IMRB-Telo15 and IMRB-Telo7 cells. *Left panel*, bulk chromatin; *right panel*, telomeres. See Figure 2A legend for details. **(C)** Quantification of (B). **(D)** qRT-PCR analyses of TERRA molecules in IMRB (blue), IMRB-Telo15 (orange) and IMRB-Telo7 (yellow) cells, normalized first to ACTB cDNA and then to IMRB (*n*=3); s.d. are shown. Note that, in IMRB-Telo7 clone, telomeric HC compaction is even higher than in IMRB-Telo15 clone, correlating with lower levels of TERRA.

**Figure S9. Telomere length analysis by TRF in SW39shATRX cell lines.** Telomere lengths of SW39 control clone ctrl-1 and three SW39 shATRX clones (shATRX-1, shATRX-2 and shATRX-3) were analyzed by TRF. Telomeres of IMRB ALT cell lines are also shown. Molecular weight (MW) marker sizes are indicated on the left (kb).

Supplementary figures
Episkopou et al.

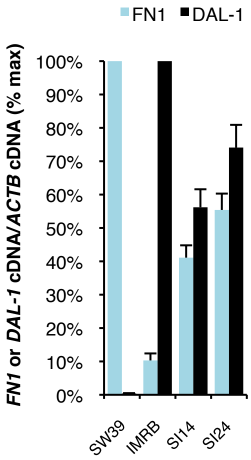
FN1
DAL-1
FN1 or DAL-1 cDNA/ACTB cDNA (% max)
100%
90%
80%
70%
60%
50%
40%
30%
20%
10%
0%
SW39
IMRB
SI14
SI24

Fig S1

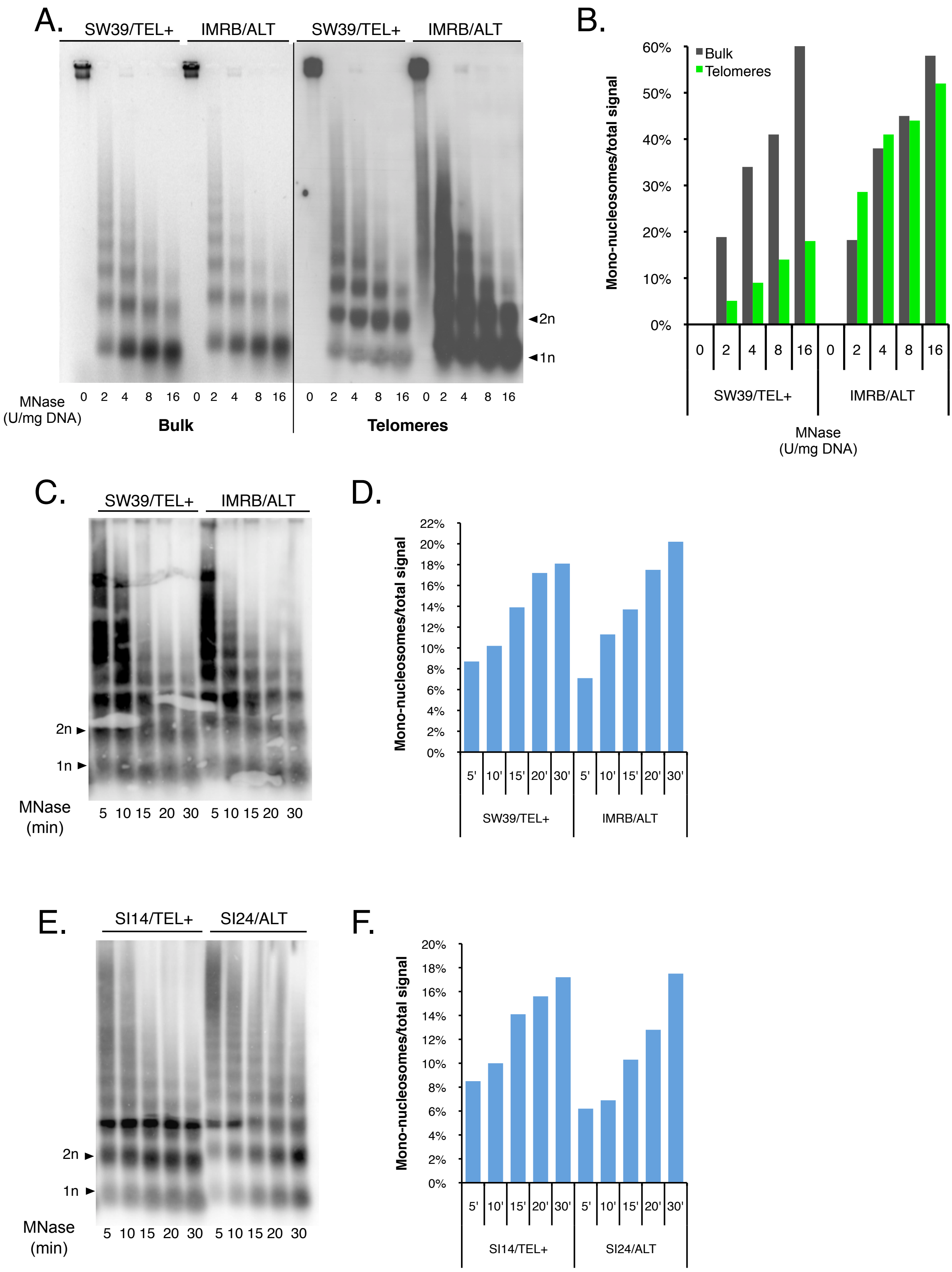
A.
SW39/TEL+
IMRB/ALT
SW39/TEL+
IMRB/ALT
◄2n
◄1n
MNase (U/mg DNA)
0 2 4 8 16 0 2 4 8 16
Bulk
0 2 4 8 16 0 2 4 8 16
Telomeres
B.
Mono-nucleosomes/total signal
60%
50%
40%
30%
20%
10%
0%
Bulk
Telomeres
0 2 4 8 16 0 2 4 8 16
SW39/TEL+
IMRB/ALT
MNase (U/mg DNA)
C.
SW39/TEL+
IMRB/ALT
2n ►
1n ►
MNase (min)
5 10 15 20 30 5 10 15 20 30
D.
Mono-nucleosomes/total signal
22%
20%
18%
16%
14%
12%
10%
8%
6%
4%
2%
0%
5' 10' 15' 20' 30' 5' 10' 15' 20' 30'
SW39/TEL+
IMRB/ALT
E.
SI14/TEL+
SI24/ALT
2n ►
1n ►
MNase (min)
5 10 15 20 30 5 10 15 20 30
F.
Mono-nucleosomes/total signal
20%
18%
16%
14%
12%
10%
8%
6%
4%
2%
0%
5' 10' 15' 20' 30' 5' 10' 15' 20' 30'
SI14/TEL+
SI24/ALT

Fig S2

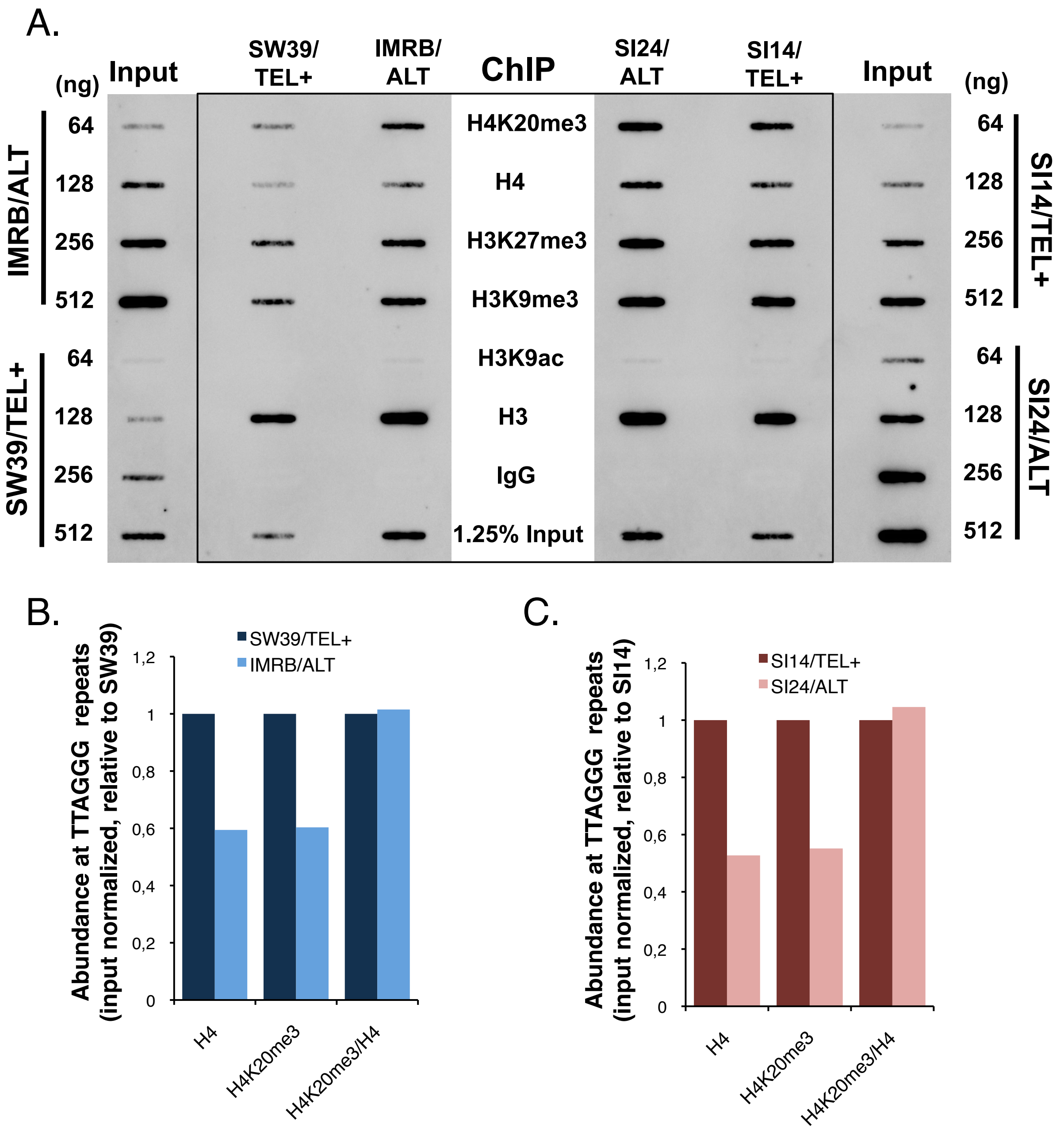
A.
(ng)
Input
SW39/ TEL+
IMRB/ ALT
ChIP
SI24/ ALT
SI14/ TEL+
Input
(ng)
IMRB/ALT
SW39/TEL+
SI14/TEL+
SI24/ALT
64
128
256
512
H4K20me3
H4
H3K27me3
H3K9me3
H3K9ac
H3
IgG
1.25% Input
B.
Abundance at TTAGGG repeats (input normalized, relative to SW39)
SW39/TEL+
IMRB/ALT
1,2
1
0,8
0,6
0,4
0,2
0
H4
H4K20me3
H4K20me3/H4
C.
Abundance at TTAGGG repeats (input normalized, relative to SI14)
SI14/TEL+
SI24/ALT
1,2
1
0,8
0,6
0,4
0,2
0
H4
H4K20me3
H4K20me3/H4

Fig S3

A.

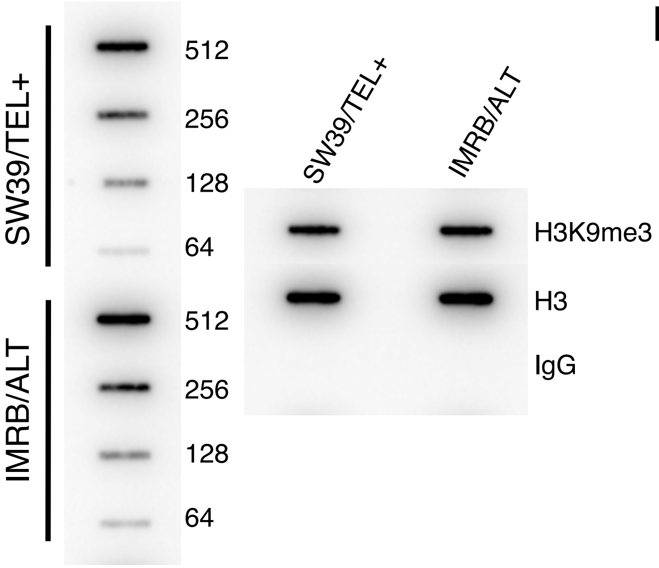
SW39/TEL+
IMRB/ALT
512
256
128
64
512
256
128
64
SW39/TEL+
IMRB/ALT
H3K9me3
H3
IgG

B.

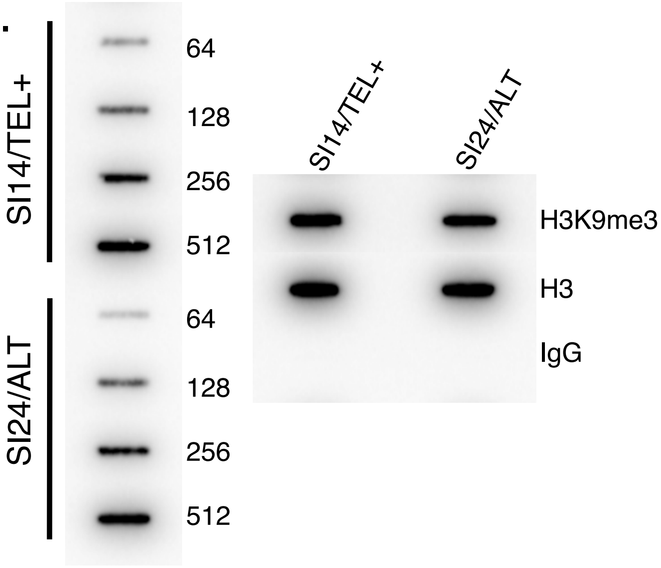
SI14/TEL+
SI24/ALT
64
128
256
512
64
128
256
512
SI14/TEL+
SI24/ALT
H3K9me3
H3
IgG

Fig S4

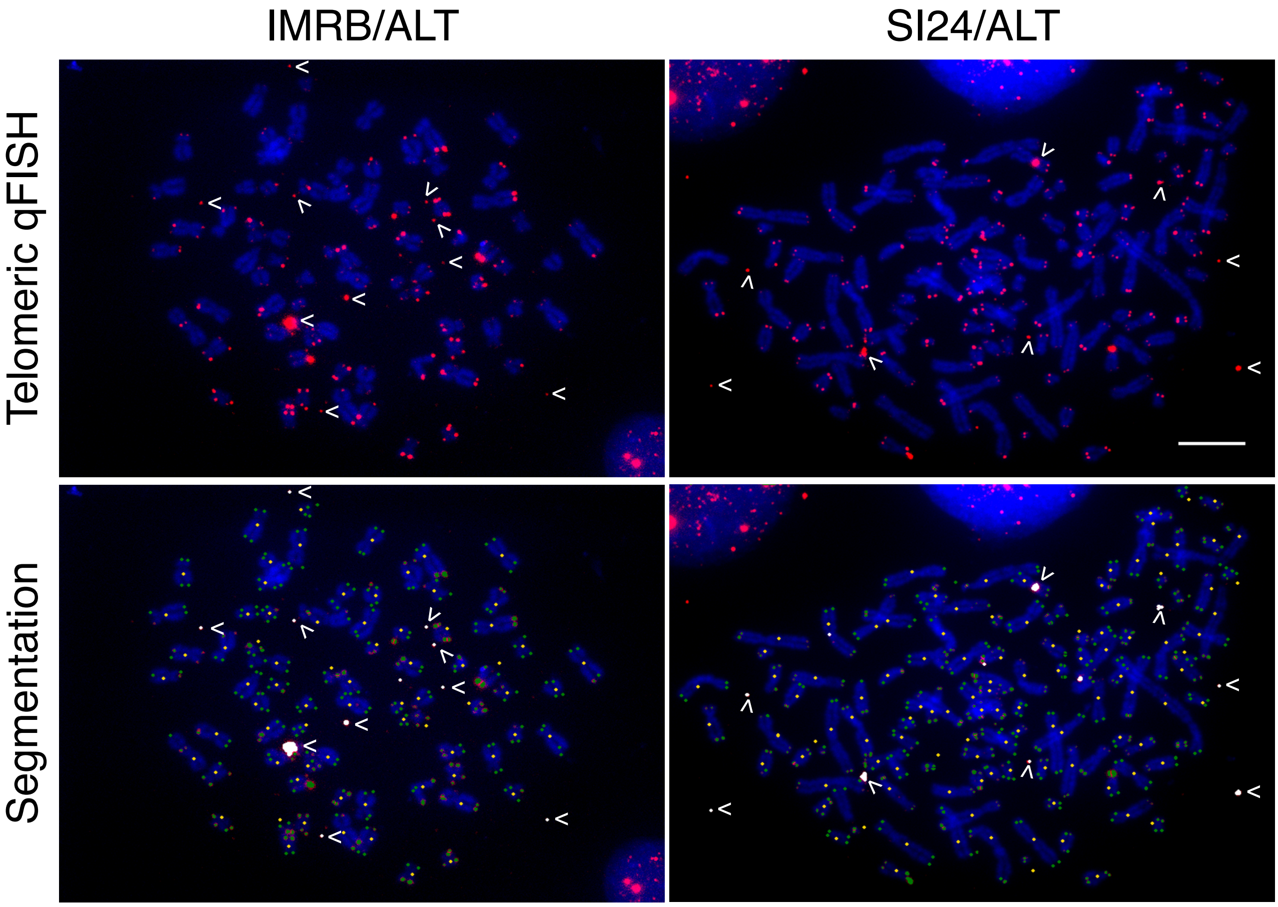


Fig S5

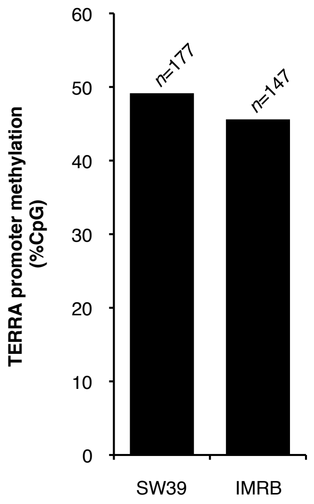
TERRA promoter methylation (%CpG)
60
50
40
30
20
10
0
n=177
n=147
SW39
IMRB

Fig S6

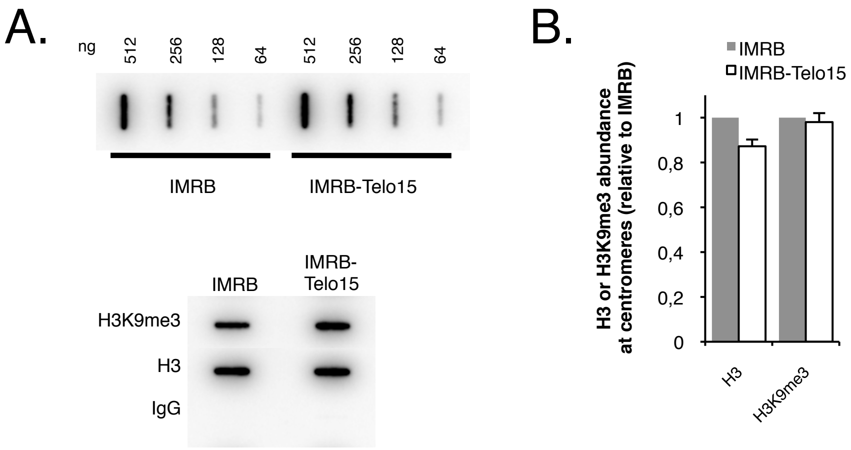

A.
ng
512
256
128
64
512
256
128
64
IMRB
IMRB-Telo15
IMRB
IMRB-Telo15
H3K9me3
H3
IgG
B.
IMRB
IMRB-Telo15
H3 or H3K9me3 abundance at centromeres (relative to IMRB)
1
0,8
0,6
0,4
0,2
0
H3
H3K9me3


Fig S7

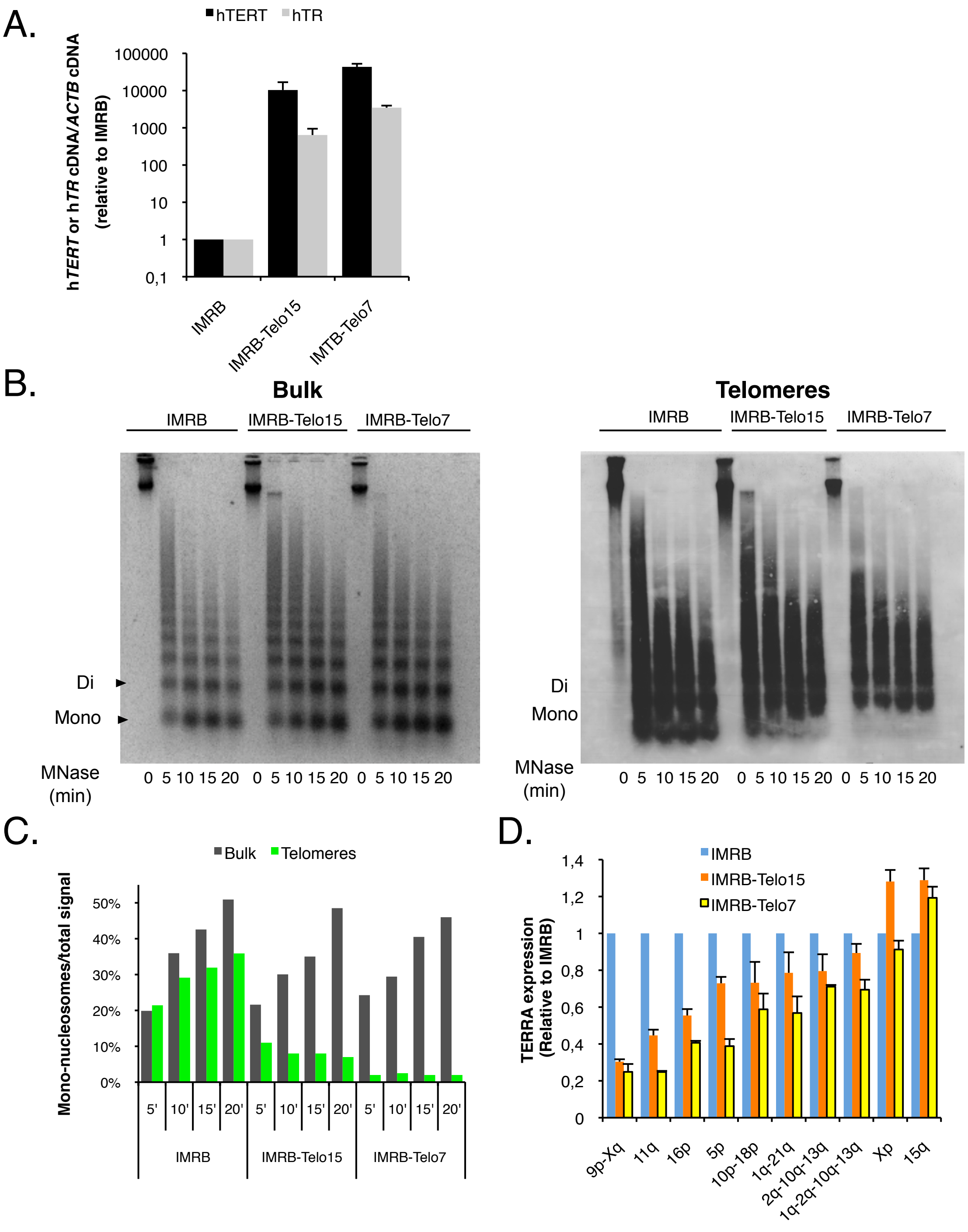
A.
hTERT
hTR
hTERT or hTR cDNA/ACTB cDNA (relative to IMRB)
100000
10000
1000
100
10
1
0,1
IMRB
IMRB-Telo15
IMTB-Telo7
B.
Bulk
IMRB
IMRB-Telo15
IMRB-Telo7
Di
Mono
MNase (min)
0 5 10 15 20 0 5 10 15 20 0 5 10 15 20
Telomeres
IMRB
IMRB-Telo15
IMRB-Telo7
Di
Mono
MNase (min)
0 5 10 15 20 0 5 10 15 20 0 5 10 15 20
C.
Bulk
Telomeres
Mono-nucleosomes/total signal
50%
40%
30%
20%
10%
0%
5' 10' 15' 20' 5' 10' 15' 20' 5' 10' 15' 20'
IMRB
IMRB-Telo15
IMRB-Telo7
D.
IMRB
IMRB-Telo15
IMRB-Telo7
TERRA expression (Relative to IMRB)
1,4
1,2
1
0,8
0,6
0,4
0,2
0
9p-Xq
11q
16p
5p
10p-18p
1q-21q
2q-10q-13q
1q-2q-10q-13q
Xp
15q

Fig S8

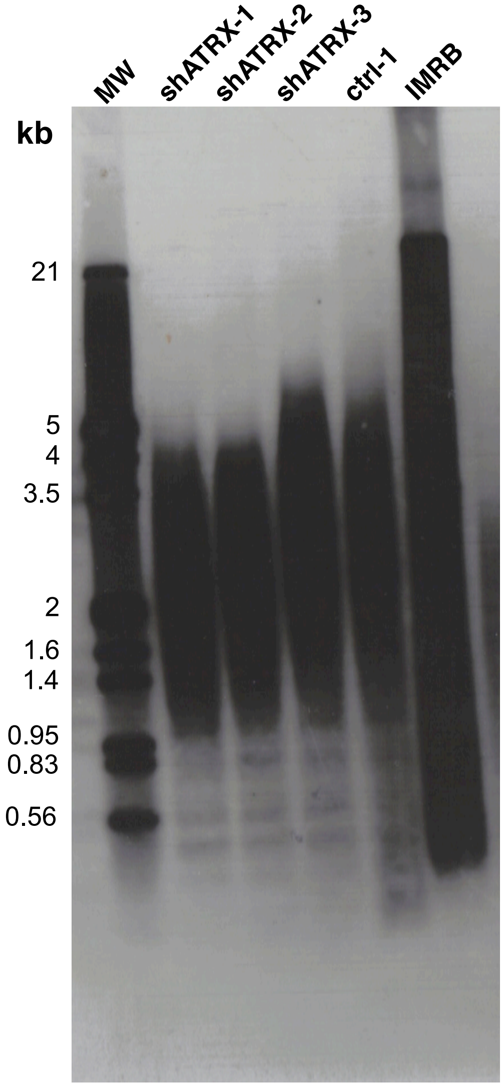
MW
shATRX-1
shATRX-2
shATRX-3
ctrl-1
IMRB
kb
21
5
4
3.5
2
1.6
1.4
0.95
0.83
0.56

Fig S9
